# Draft Genome Sequence of *Agrobacterium rhizogenes* Strain NCPPB2659

**DOI:** 10.1128/genomeA.00746-16

**Published:** 2016-07-28

**Authors:** Jose A. Valdes Franco, Ray Collier, Yi Wang, Naxin Huo, Yong Gu, Roger Thilmony, James G. Thomson

**Affiliations:** a USDA-ARS Crop Improvement and Genetics, Western Regional Research Center, Albany, California, USA; b Department of Plant Sciences, University of California, Davis, Davis, California, USA

## Abstract

This work reports the draft genome sequence of *Agrobacterium rhizogenes* strain NCPPB2659 (also known as strain K599). The assembled genome contains 5,277,347 bp, composed of one circular chromosome, the pRi2659 virulence plasmid, and 17 scaffolds pertaining to the linear chromosome. The wild-type strain causes hairy root disease in dicots and has been used to make transgenic hairy root cultures and composite plants (nontransgenic shoots with transgenic roots). Disarmed variants of the strain have been used to produce stable transgenic monocot and dicot plants.

## GENOME ANNOUNCEMENT

*Agrobacterium rhizogenes* is a Gram-negative soil bacterial plant pathogen that causes hairy root disease (1) resulting from *in planta* expression of *rol* genes (2) after transfer DNA (T-DNA) movement into the host plant cells (3). The Ri T-DNA of *A. rhizogenes* strain NCPPB2659, isolated from cucumbers displaying hairy root disease, was characterized (4), and the pRi2659 virulence plasmid sequenced (5). *A. rhizogenes* strain NCPPB2659 induces hairy roots from transformation-recalcitrant plants, like soybean, with higher efficiency relative to other strains (6), and this characteristic has been used for gene function studies in hairy root cultures (7, 8) and composite plants (9). The strain has been disarmed by two independent groups (5, 10) and used to generate stable transgenic monocot (5, 11) and dicot (12) plants. To enhance the stability of constructs containing large synthetic T-DNA molecules in the wild-type and disarmed strains (such as *A. rhizogenes* 18r12v), the genome sequence data were used to design a cassette for the *TcR* insertional inactivation of *recA* (13, 14). These variant strains with deactivated *recA* remain competent for plant transformation (data not shown).

Genomic and plasmid DNA were extracted from *A. rhizogenes* strain NCPPB2659, according to the protocol of Wise et al. (15). Illumina paired-end libraries were constructed from sonicated DNA using the Kapa DNA library preparation kit (Kapa Biosystems). Fragments between 500 and 1,000 bp were selected with a polyethylene glycol (PEG)-NaCl solution method (Kapa Biosystems). A total of 3,165,594 paired-end reads were generated on an Illumina MiSeq, at a read length of 300 bp. Reads were error corrected with Quake version 0.3.5 (16) and quality filtered and trimmed with Trimmomatic version 0.32 (17), resulting in 2,546,326 high-quality reads. The initial sequence assembly was performed using SOAP*denovo*2 (18). This assembly produced 25 scaffolds (minimum, 617 bp; maximum, 1,934,317 bp; *N*_50_, 1,468,096 bp). Due to the fragmented nature of the resulting assembly, a manual analysis was performed on the CLC Genomics Workbench version 8.5 (CLC bio/Qiagen) to reveal low-coverage regions and broken paired-end reads and identify collapsed or missing regions, within and between the scaffolds. These regions were then PCR amplified and characterized by sequencing. Alignment analysis of these sequences with the scaffolds allowed assembly of the complete circular chromosome. Subsequent mapping analyses validated the assembly (>97% of the reads mapped in pairs with uniquely assigned matches, excluding clearly repetitive areas showing higher-than-average coverage). This allowed the generation of a final collection of 18 sequences (*N*_50_, 3,003,247 bp), with a complete circular chromosome and 17 scaffolds of proposed linear chromosome (excluding the prior published virulence plasmid sequence), composed of 5,277,347 bp, with a G+C content of 59.8% and an overall coverage of approximately 110×.

Automated annotation was performed by the NCBI Prokaryotic Genome Annotation Pipeline. *A. rhizogenes* strain NCPPB2659 contains 4,860 predicted coding sequences, 72 predicted RNA genes, and 4 complete copies of each rRNA. The 33 identified pseudogenes exhibit no obvious sequencing or assembly mistakes.

### Nucleotide sequence accession numbers.

The entire genome shotgun sequence data set has been deposited at DDBJ/EMBL/GenBank under accession no. LYBK00000000. Illumina raw reads are available at the NCBI Short Read Archive under accession no. SRR3537230.
